# Efficacy of tocilizumab for refractory Takayasu arteritis: a retrospective study and literature review

**DOI:** 10.1007/s00380-021-01981-1

**Published:** 2021-11-08

**Authors:** Haiyan Li, Zongwen Shuai

**Affiliations:** Department of Rheumatology and Immunology, The First Affiliated Hospital of Anhui Medical University, Hefei, 230022 China

**Keywords:** Takayasu arteritis, Tocilzumab, Interleukin-6, C-reactive protein, Erythrocyte sedimentation rate

## Abstract

To evaluate the efficacy and safety of tocilizumab (TCZ) in the treatment of refractory Takayasu arteritis (TAK). Eleven refractory TAK patients treated with TCZ at the First Affiliated Hospital of Anhui Medical University between 2017 July and 2020 December were respectively analyzed. We also respectively analyzed the studies on TCZ efficacy in patients with TAK, from PubMed/MEDLINE, Elsevier Science Direct between January 2010 and April 2021. The median age of 11 patients was 34(19–46) years. After 3 months of TCZ, a significant drop was found in median NIH (3[2–5] at baseline vs 1[0–2] after 6 months; *p* < 0.05), ITAS-2010 score (8.5[6–11] vs 6[1–10]; *p* < 0.05). One (9%) patient experienced relapse during TCZ treatment. After withdrawal of TCZ, one patient (9%) underwent relapse and nine patients (81%) were spared of GC use. In literature review, a total of 211 patients (mean age 35 years) were analyzed, including 80 (38%) Chinese and 169 females (80%). Among the 211 patients, (154 patients) 73% achieved remission after the last infusion of TCZ; TAK relapsed in 6% of patients during TCZ treatment and 5% of the TCZ patients after the withdrawal of TCZ. A total of 95 types of adverse events were observed in the literature. Infection was the most common adverse effect, occurring in 50% of patients. TCZ could serve as an efficacious and safe agent for refractory TAK.

## Introduction

Takayasu arteritis (TAK) is a major large vessel vasculitis involving the aorta, its major branches and the pulmonary arteries [1]. TAK is more prevalent in Asia and the Middle East than in other regions [2]. TAK predominantly affects the young women in their twenties or thirties [3]. Without early effective anti-inflammatory treatments, TAK patients may undergo relapse after continuous inflammation and vascular injury [4].

The pathogenesis of TAK remains unclear. Multiple studies have revealed the active roles of pro-inflammatory cytokines, including tumor necrosis factor α (TNFα) and interleukin-6 (IL-6). IL-6 level increases in the serum, so does its expression in the aorta [5, 6]. In addition, IL-6 can mark the activity of various diseases [7]. Therefore, baseline IL-6 level has been suggested to predict TAK relapse during a long-term follow-up [8]. Blockade of IL-6 signaling may counter TAK, which has been testified in experimental or clinical studies [9].

Glucocorticoids (GCs) can induce remission in almost 60% of TAK patients [10]. However, their long-term use results in various side effects, and brings with a high rate of relapse, even the dosage is tapered off gradually [11]. To overcome these events, traditional immunosuppressive agents are recommended [12]. However, those patients with a high disease activity may not react sufficiently.

Tocilizumab (TCZ), a humanized monoclonal antibody targeting the IL-6 receptor subunit alpha (IL-6Ra), has demonstrated a significant efficacy against rheumatoid arthritis. A number of observational studies and randomized control trials (RCTs) have reported that TCZ renders clinical improvement and curbs TAK progression [13]. More importantly, TCZ could alter the mural thickness of arteries affected, thereby facilitating the reduction of steroid dose [14, 15].

We report herein a retrospective study and a literature review on the efficacy and tolerance of TCZ in Chinese patients.

## Methods

### Patients and methods

#### Patients

This was a retrospective single-center study based on patients aged 16 or more at the moment of informed consent and recruited from the First Affiliated Hospital of Anhui Medical University between July 2017 and December 2020. The diagnosis was established according to the Indian Takayasu clinical activity score (ITAS2010). The data of patients’ clinical manifestations, laboratory indexes, imaging features and treatment outcomes at TCZ initiation, 3, 6 and 12 months were recorded. The dosage of GCs was 8 mg/kg (once a month, intravenous infusion).

#### Clinical efficacy assessment and definition

The clinical efficacy was determined as favorable changes in symptoms and signs, GC dosage and imaging features after TCZ treatment. Remission was defined as lack of clinical manifestations of active disease and daily prednisone dosage less than 10 mg/day with a NIH < 2. Relapse was defined as a condition, in which remission had been achieved, but disease turned active again, and treatment was needed.

### Search strategy

From PubMed/MEDLINE, Elsevier Science Direct, we searched studies about TCZ in the treatment of TAK. All articles were limited to full text, English language, and publication date between January 2010 and April 2021. The keywords included TAK, TCZ, inerleukin-6/IL-6. Studies with patients aged < 16 years and case reports with less than five subjects were excluded.

### Retrospective analysis

All the data were collected from outpatient and inpatient medical records, including clinical manifestations, blood biochemical indexes and follow-up information was compared with our reports (2021), Abisror et al. [16], Goel et al. [17], Tombetti et al. [18], Canas et al. [19], Mekinian et al. [20], Loricera et al. [21], Zhou et al. [15], Mekinian et al. [12], Nakaoka et al. [13], Kilic et al. [22], Kong et al. [23], Pan et al. [24], Wu et al. [25] research group.

### Statistical analysis

Continuous variables were presented as medians with ranges, and qualitative variables as frequencies with percentages. Fisher’s exact test was carried out to compare qualitative variables. We used independent samples *t* test to analyze continuous variables with normal distribution and the Mann–Whitney or Wilcoxon test to compare continuous variables with non-normal distribution. As appropriate. *p* < 0.05 was considered as statistically significant.

## Results

### Patient characteristics

**Fig. 1:**
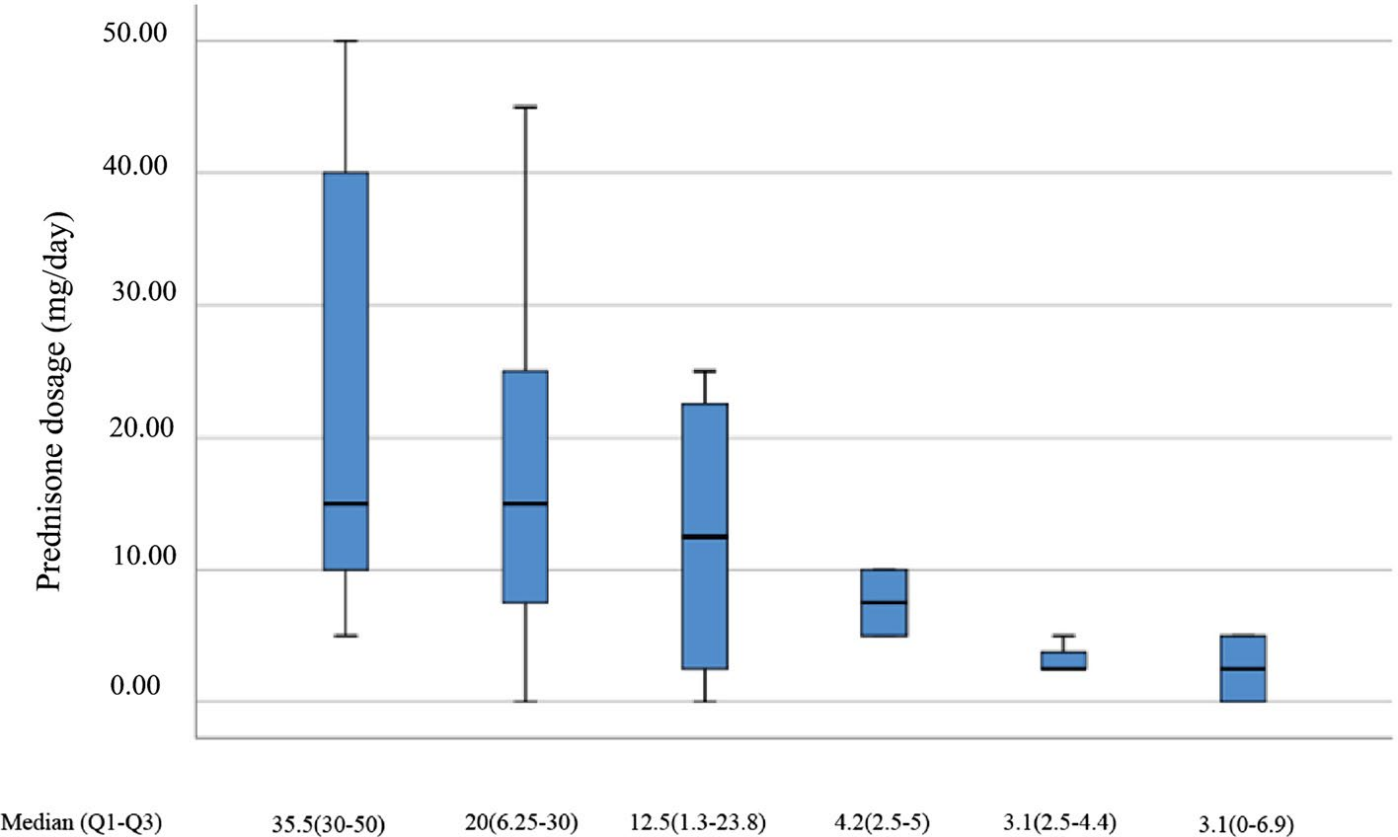
Daily prednisone dosage during a 12-month follow-up

A total of patients (one male, ten females) with TAK were included in the study. The mean age at disease onset was 34 years (ranges 19–46 years), similar to that reported in other TAK patients. The average duration of TAK before TCZ treatment was 32.3 months. Three patients discontinued TCZ after first infusion. Two patients refused TCZ due to high cost and one for neutropenia and pneumonia. The main parameters about disease activity are described in Table 1. The levels of acute inflammatory markers showed a remarkable decrease. After TCZ therapy for 6 months, C-reactive protein (CRP) decreased from 34.1 mg/L (13.3–55.1) to 0.6 mg/L (0.3–0.9), erythrocyte sedimentation rate (ESR) from 41.6 mm/h (29–49) to 10.5 mm/1 h (4.3–15.3). NIH scores declined significantly from 3 (2–5) to 1 (0–2) (*p* < 0.001), ITAS2010 baseline from 7 (6–9) to 3 (1–5) (*p* < 0.001). After a 6-month TCZ treatment, the dosage of GCs was reduced from 35.5 mg (30–50) to 3.1 mg (0–6.9) (Fig. 1). Eight patients (72.7%) achieved remission after six infusions of TCZ. Six patients (54.5%) developed vascular bruits. Four patients (36.4%) showed pulse weakness. Two patients showed a change of over 10 mmHg in blood pressure. Among the vascular manifestations in different studies, vascular bruits accounted for the biggest proportions (54.5%, 100%, 100%. 92.6%, respectively).

**Table 1:** Patients’ characteristics at initiation of TCZ and during follow-up

	At initiation of TCZ	At 3 months	At 6 months	At 12 months
Patients treated with TCZ (%(*n*))	11(100)	8(72.7)	8(72.7)	3(27.3)
NIH score	3	2	1*	1
ITAS 2010	7	3	3*	2
Remission (%)	0	5(45.5)	8(72.7)	3(27.3)
CRP	34.1	0.6*	0.6*	1.6*
ESR	41.6	5.5*	8.0*	10.5*
Prednisone (mg/day)	35.5	4.2*	3.1*	3.1*
Immunosuppressive drugs	AZP (*n* = 2); CTX (*n* = 3); MTX (*n* = 2); MMF (*n* = 2); TK506 (*n* = 1)	0	0	0
WBC (10^9^/L)	8.4	10.7	9.74	8.2
NE (10^9^/L)	5.1	5.7	6.0	5.4
LY (10^9^/L)	2.3	3.3	3.0	2.2
PLT (10^12^/L)	268.2	268.0	261.0	237.5
NLR	2.6	1.7	2.1	3.5
PLR	139.7	80.9	88.7	106.8
RBC (10^12^/L)	4.3	4.3	4.4	4.4
Hb (g/L)	115	122	121	139
ALT (U/L)	19.1	16.5	19.3	18
Cr (μmol/L)	74.6	55	55.3	58.8
Glu (mmol/L)	4.6	4.5	4.7	5.1

*ALT* alanine aminotransferase;* Cr* creatinine;* CRP* C-reactive protein; *ESR* erythrocyte sedimentation rate; *Glu* glucose; *Hb* hemoglobin; *LY* lymphocyte; *NE*, neutrophil; *NLR*, neutrophil-to-lymphocyte ratio; *PLT* platelets; *PLR* platelet-to-lymphocyte ratio; *RBC* red blood cell; *WBC* white blood cell

*p < 0.05

### Outcomes between post-treatment 6 months and 12 months

Among the 8 patients, 3 patients (27%) continued to receive a total of 12 infusions. The other five patients (45%) received 6 months of TCZ and then GCs without TCZ. During the 12-month follow-up, seven patients (64%) were treated with only steroids, and one patient (9%) was not exposed to any other treatment (Table 1). All eight patients displayed no relapse.

### Literature review

A total of 211 patients (mean age 35 years) were reviewed, including 80 (38%) Chinese and 169 females (80%). Their demographic characteristics and clinical manifestations are concluded in Table 2. For Chinese patients, the median duration from disease onset to TCZ treatment was 12 (6–12) months, shorter than that reported in other populations (mean 16 months, ranges 11–24 months). Overall, type V vasculitis (45/110, 40.9%) was the most common type among all populations, followed by Type. In China, type I topped others (25/71, 35.2%), followed by type V (24/71, 33.1%) based on the 1996 Numano classification. However, in other populations, type V accounted for the highest proportion (21/39, 54%), followed by type II (8/39, 20.5%). Vascular bruits were found in 67 (36.2%), constitutional symptoms in 12 (6.5%), limb claudication in 27 (14.6%), neurological symptoms in 23 (12.4%), pulse weakness in 32 (17.3%), abnormal blood pressure in 24 (13%) patients. In China, vascular bruits were found in 31 (38.8%), constitutional symptoms in 12 (15%), limb claudication in 11 (13.8%), neurological symptoms in 21 (26.3%), and pulse weakness in 45 (39%) patients. Before the use of TCZ, more than half of the whole populations had received traditional immunosuppressive agents. MTX (47.4%) was the most frequently used. Prior to TCZ therapy, anti-TNFα was the most common, found in 89% (22/25) of all agents.

**Table 2:** Main features of the onset before Tocilizumab therapy in patients with refractory Takayasu arteritis in  literature

Variables	This study	Abisror [16]	Goel [17]	Tombetti [18]	Canas [19]	Mekinian [20]	Loricera et al. [21]	Zhou et al. [15]	Mekinian[12]	Nakaoka [13]	Kilic [22]	Kong et al. [23]	Pan et al. [24]	Wu et al. [19]
*n*	11	5	10	7	8	14	8	16	46	18	15	9	11	33
Year	2018	2013	2013	2013	2014	2015	2016	2017	2018	2018	2020	2018	2020	2021
Female (%(*n*))	82(9)	80(5)	90(9)	100(7)	100(8)	64(7)	100(8)	94(15)	76(35)	88.9(16)	86.7(13)	89(8)	91(10)	82(27)
Age	32.3	54	24.5	24	31	42	34	33.1	43	31.1	35	32.1	35.6	26
Treatment duration prior TCZ (months)	12	–	25.5	14	18		11	12	–	12	24	6	6	14
Numano subtype (%(*n*))														
I	54.5(6)	–	30(3)	–	12.5(1)	–	–	43.7(7)	–	11.1(2)	–	–	11.1(2)	30(10)
II	9.1(1)	40(2)		–	12.5(1)	–	–	25(4)	–	27.8(5)	–	–	22.2(4)	24(8)
III	0	–		–	–	–	–	0	–	16.7(3)	–	–	0	3(1)
IV	18.2(2)	–		–	12.5(1)	–	–	6.3(1)	–	0	–	–	0	3(1)
V	18.2(2)	20(1)	70(7)	–	12.5(1)	–	–	25(4)	–	44.4(8)	–	–	45.5(5)	39(13)
Constitutional symptoms (%(*n*))	27.3(3)			–	100(8)	64(7)	50(4)	56.3(9)	37.2(16)	–	–	–	–	–
Limb claudication (%(*n*))	18.2(2)	–	–	–	12.5(1)	–	12.5(1)	43.8(7)	30.5(14)	–	–	22.2(2)	–	–
Neurological symptoms (%(*n*))	0	–	–	–	25(2)	–	–	18.8(3)	–	–	–	33.3(3)	–	45(15)
Vascular bruits (%(*n*))	54.5(6)	–	–	–	50(4)	–	–	100(16)	70(32)	–	–	–	–	27(9)
Pulse weakness (%(*n*))	36.4(4)	20(1)	–	–	75(6)	–	–	68.8(11)	–	–	–	11.1(1)	–	30(10)
Blood pressure differences > 10 mmHg(%(*n*))	18.2(2)	20(1)	–	–	50(4)	–	–	31.3(5)	–	–	–	22.2(2)	100(11)	–
Immunosupressive aents before TCZ	AZP (*n* = 2); CTX (*n* = 3); MTX (*n* = 2); MMF (*n* = 2); Tac (*n* = 1); Tripterygium glycosides (*n* = 1); Thalidomide (*n* = 1)	AZP (*n* = 2) CTX (*n* = 1) MTX (*n* = 3)	AZP (*n* = 2) MMF (*n* = 8) MTX (*n* = 1)	AZP (*n* = 4) CYC (*n* = 3) CTX (*n* = 1) MTX (*n* = 7) SIR (*n* = 2)	AZP (*n* = 4) CYC (*n* = 2) MTX (*n* = 4)	–	AZP (*n* = 2) CTX (*n* = 2) CYC (*n* = 1) MMF (*n* = 2) MTX (*n* = 5)	CTX(*n* = 12); MMF (*n* = 9)	MTX = 13	–	AZP (*n* = 1) CYC (*n* = 8) MMF (*n* = 2) MTX (*n* = 12) LEF (*n* = 1) SSZ (*n* = 2)	–	MTX(*n* = 6)	LEF (*n* = 9), MTX (*n* = 5), SIR (*n* = 1)
Biologic agents	IFX (*n* = 1)	IFX (*n* = 1)	–	IFX (*n* = 1) adalimumab (*n* = 3) anakinra and rituximab (*n* = 1)	IFX (*n* = 1)	–	IFX (*n* = 4) ADA (*n* = 2)	TNFα antagonusts (*n* = 2)	TNFα antagonusts (*n* = 5) RTX (*n* = 1)	–	ADA (*n* = 2) ETN (*n* = 1) IFX (*n* = 2)	–	–	–

*AZP* Azathioprine; *CYC* cyclosporine; *CTX* cyclophosphamide; *LEF* Leflunomide; *MTX* Methotrexate; *MMF* Mycophenolate mofetil; *SIR* sirolimus; *SSZ* sulfasalazine; *Tac* Tacrolimus; *IFX* Infliximab

The already-reported responses to TCZ are summarized in Table 3. Among the 211 patients reviewed, 154 (73%) patients achieved clinical remission after TCZ treatment. Clinical remission was achieved in 82.5% of Chinese patients, and 67.2% in overseas patients, though assessment criteria of disease activity were different. The median TCZ duration was 7.9 (6–18) months. Among the 211 patients, (154 patients) 73% achieved remission after the last infusion of TCZ; TAK relapsed in 6% of patients during TCZ treatment and 5% of the TCZ patients after the withdrawal of TCZ. In China, only one (1.2%) patient experienced relapse during TCZ treatment, significantly fewer than those overseas (*n* = 4, *p* = 0.026). After withdrawal of TCZ, seven (8.8%) patients showed a relapse. All the studies in China revealed that the use of TCZ led to a significant reduction in the dosage of GCs. Most importantly, the percentage of TAK patients with relapse during TCZ treatment in our study was significantly lower than that in overseas studies.

**Table 3:** Response to Tocilizumab therapy and changes of clinical parameter in  literature

Characteristics	This study	Abisror [16]	Goel [17]	Tombetti [18]	Canas [19]	Mekinian [20]	Loricera [21]	Zhou et al. [15]	Mekinian [12]	Nakaoka [13]	Kilic [22]	Pan et al. [23]	Kong et al. [24]	Wu et al. [25]
Median duration TCZ (months)	6	–	7.9	14	18	6	15.5	24.5	18	14	15	6	6	6
Remission on TCZ (%(*n*))	73(8)	50(2)	90(9)	57(4)	100(8)	50(7)	87.5(7)	81(13)	89(41)	55.6(10)	87(13)	100(11)	100(9)	70(23)
Relapse during therapy (%(*n*))	0(0)	20(1)	0	57(4)*	0(0)	–	–	0(0)	–	44(8)*	13 (2)	–	0(0)	9(1)
Relapse after withdrawal of TCZ (%(*n*))	9.1(1)	–	30(3)	–	–	–	12.5(1)	–	–	–	–	–	–	43(6)
Steroid-sparing (%(*n*))	9.1(1)	20(1)	10(1)	–	–	–		6(1)	-	-	-	45(5)	–	–
Kerr score (before/after)	3/0	–	–	–	–	–	–	–	–	–	–	2/0	8/1	–
NIH (before/after)	3/1	–	–	–	–	–	–	–	3/0	–	–	–	–	2/2
ITAS2010 (before/after)	7/3	–	4/1	–	–	–	–	–	–	–	–	9/1	5.67/2.67	–
Prednisone (mg/day) (before/after)	35.5/4.2	–	24/5.4	10/6.2	43.8/6.9	12.5/10	42.5/2.5	24.8/7.9	15/4	–	16.2/7.1	7.5/2.5	30.0/10.0	30.0/15
CRP (mg/L) (before/after)	34.1/0.6	14.9/8.0	–	13/2	2.3/1.0	24/2	3.1/0.2	28.9/0.6	23/1	–	39.8/7.9	3.2/0.72	53.3/12.7	11.2/0.9
ESR (mm/h) (before/after)	41.6/5.5	36.7/6.9	–	34/4	39.8/13.1	–	40/3	39/6	–	–	26/3	13/2	73.9/9.4	41.0 /4.0
WBC (10^9^/L) before/after)	8.4/8.2	–	–	9.25/9.01	–	–	–	10.0/8.1	–	–	–	7.6/7.5	10.5/9.3	–
NE (10^9^/L) (before/after)	5.1/5.0	–	–	5.53/5.60	–	–	–	–	–	–	–	4.7/4.6	–	–
LY (10^9^/L) (before/after)	2.3/2.4	–	–	2.99/2.63	–	–	–	–	–	–	–	2.4/2.4	2.9/3.2	–
PLT (10^12^/L) (before/after)	268.2/237.5	–	–	370/277	–	–	–	–	–	–	–	259.7/222.6	360.0/253.6	–
NLR (before/after)	2.6/3.5	–	–	1.9/2.2	–	–	–	–	–	–	–	2.0/1.9	–	–
PLR (before/after)	139.7/106.8	–	–	123.7/105.3	–	–	–	–	–	–	–	108.2/92.8	124.1/79.3	–
RBC (10^12^/L) (before/after)	4.3/4.4	–	–	–	–	–	–	–	–	–	–	4.4/3.4	–	–
Hb (g/L) (before/after)	115/139	–	–	105/110	–	–	–	–	–	–	–	128.6/138.4	–	–

*CRP* C-reactive protein; *ESR* erythrocyte sedimentation rate; *RBC* red blood cell; *Hb*, hemoglobin; *WBC* white blood cell; *NE* neutrophil; *LY* lymphocyte; *PLT* platelets; *NLR* neutrophil-to-lymphocyte ratio; *PLR* platelet-to-lymphocyte ratio

*p < 0.05

The laboratory parameters are summarized in Table 3. The levels of acute phase inflammation markers, including CRP and ESR, varied a lot among all patients. Additionally, after TCZ treatment, the counts of white blood cells (WBs) and neutrophils (NEs) decreased, but the counts of lymphocytes (LYs), red blood cell (RBCs), platelets (PLTs) and hemoglobin increased, as compared with those before TCZ treatment.

### Safety

**Fig. 2:**
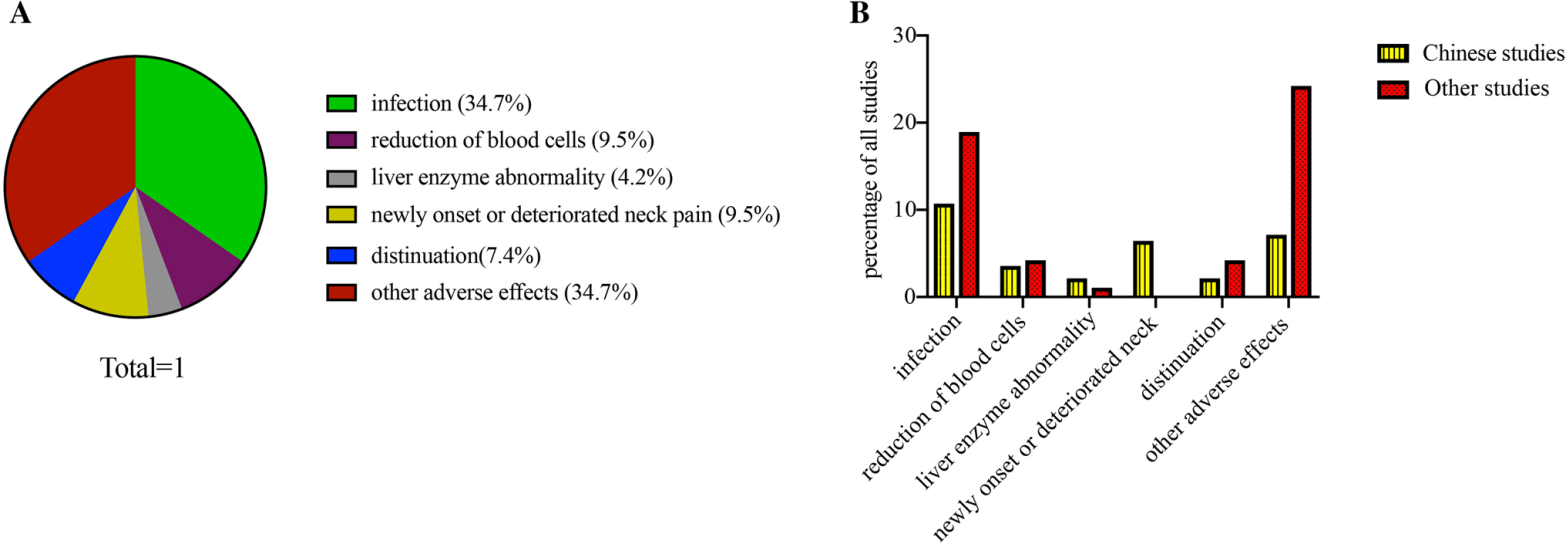
Adverse events reported in the literature; types and proportions of infections in the literature. **A** Proportions of adverse events in the literature. **B** Types and proportions of infections in the literature

One patient (9.1%) withdrew TCZ because of leukopenia and pulmonary infection in our study. A total of 95 types of adverse events were reported in the literature. Infection was the most common adverse effect, observed in 50% of patients. Liver enzyme abnormality was the least common adverse effect. Newly diagnosed or deteriorated neck pain was the second most common adverse event in Chinese patients (20%). Other adverse effects included skin rash, liver enzyme abnormality, gastro-intestinal disorders, and neoplasms. No serious adverse effects and death were observed. Figure 2 shows the adverse effects in other studies and ours.

## Discussion

Our study clarified the potential efficacy of TCZ in Chinese patients with refractory TAK in the active phase. During the 6-month follow-up, TCZ significantly decreased the ESR, CRP, and NIH scores as well as ITAS 2010 in patients with TAK. The present study is possibly the first to elegantly describe the characteristic of global TAK patients and the efficiency of TCZ using the diagnostic standard in 1990 American College of Rheumatology (ACR). Consistent with previous overseas studies, the present study held that TAK mainly attacks the young female patients [26]. Types I and V showed up in more than 50% of Chinese TAK patients. In other countries, types II and V were the most common. In either China or other countries, the vascular bruits are the most common vascular sign.

In our study, TCZ was used as an optional therapy for those patients who failed to adequately respond to GCs and immunosuppressive agents (100%) or anti-TNF agents (9.1%). Patients with TAK took the GCs combined with disease-modifying rheumatic drugs (DMARDS) as the first-line strategy. CTX and MTX were the most common DMARDs (in 27.8% and 24.1%, respectively) to be combined with GCs. However, Ohigashi has reported that more than 50% of TAK patients treated with GC mono-therapy suffered from disease flare during GC dosage tapering [27]. Considering the high rate of relapse after GC therapy, immunosuppressive agents and/or biological agents are supplemented to provide more benefits. In our analysis, 45 TAK patients lacking clinical response to GCs and anti-TNF agents benefited from TCZ therapy.

The management of TAK has not been standardized. One study has shown that TCZ and anti-TNFα agents triggered similar partial and complete responses at post-treatment 3, 6 and 12 months [12]. Our study demonstrated that TCZ was usually prescribed as an optional drug after traditional immunosuppressive or anti-TNFα agents failed to produce desirable effects. Anti-TNFα agents are used as the first choice, but cannot induce remission in more than 10% of patients. However, a 6-month treatment of TCZ realized clinical remission in our analysis. Tombetti has reported the similar results in seven patients with refractory TAK from a single center [18]. Moreover, Shuai et al. found that TCZ, compared to anti-TNFα agents, achieved a higher remission rate and a lower relapse rate (70% vs 65%, 17% vs 20%, respectively) [28].

The efficacy of TCZ in treating newly diagnosed TAK patients or refractory TAK patients has been explored in European randomized trials and prospective trials [26, 29]. As shown in our review, all the studies reported significant improvement in disease activity (Kerr score or NIH or ITAS2010) as well as the laboratory parameters (mainly CRP and ESR). In TAK management guidelines, inflammatory markers are recommended as tools to monitor disease activity [30]. In the presence of TCZ, the dosage of GCs can be reduced [31]. TCZ can also increase the event-free survival rate [12]. In the present study, one patient maintained remission, although GCs dosage was reduced after a 6-month treatment of TCZ. Lo Gullo et al. also reported that a TAK patients resistant to traditional immunosuppressive drugs achieved a steroid-sparing condition through subcutaneous administration of TCZ [32], suggesting that TCZ can reduce the dosage of traditional immunosuppressive drugs, while maintain the remission in TAK patients. Our study also supported that after withdrawal of TCZ; the disease activity could be well controlled [14]. The only prospective study of TAK reported a high rate of remission of 85% [26]. In contrast, the remission rate (73%) in our study was significantly lower, probably because three patients discontinued TCZ use due to its high cost. TCZ induced complete clinical remission in 86% of Chinese patients, a rate close to that reported in a multicenter retrospective study [12]. In a previous study, the remission rate in DMARDs-treated patients was lower than that in naïve-treated patients, but their relapse rates were similar. In the present study, the relapse rate during TCZ treatment was much lower than that reported in the prospective study.

IL-6 is actively involved in the pathology of anemia of patients with TAK [15]. Zhang et al. reported that anemia was more likely to occur in young or female TAK patients with high disease activity [33]. The present study also supported that the level of hemoglobin increases with the reduction of acute inflammatory markers in TAK patients under remission.

Several studies of rheumatic disease revealed the close relationship of disease activity with platelet-to-lymphocyte ratio (PLR) as well as neutrophil-to-lymphocyte ratio (NLR) [34–36]. However, scant literature has analyzed the link between PLR/NLR and TCZ efficacy. This link was illustrated in the present study. Pan et al. suggested that a higher NLR indicated a higher disease activity in patients with TAK [35]. In the literature analysis, TAK patients showed decreased counts of leucocytes, NE and PLT, and increased counts of lymphocytes. NLR and PLR declined after the patients achieved remission, though without obvious discrepancies between Chinese and other populations. The pathological mechanism of TAK may involve the recruitment and infiltration of neutrophils in the aorta following arterial inflammation [37].

In our literature, 11 (5%) patients received 18F-FDG PET/CT imaging to assess the disease activity of TAK. However, in China, 18F-FDG-PET was not extensively recommended to TAK patients because of high fees of 18F-FDG-PET. Recent studies recommended the 18F-FDG-PET for monitoring the disease activity of TAK including assessment of the recurrence of TAK, since 18F-FDG-PET showed the simultaneous changes of inflammation in arterial walls and clinical course under the therapy of TCZ [38, 39]. In summary, FDG-PET is a promising checking method to aid the clinical evaluation of disease activity.

The safety of TCZ has been validated in various autoimmune diseases [40–42]. In our study, neutropenia and severe pneumonia were observed in one patient (9.1%) and this patient discontinued the use of TCZ because of adverse effects. In our study, adverse events were found in 32.5% of Chinese TAK patients, with severe infections accounting for 16%. These infections may arise from leukopenia and neutropenia. Additionally, high-dose or continuous GCs treatment may increase the risk of recurrent infections by bacteria or fungus, such as invasive Aspergillosis [43]. In our study, only one patient suffered from pulmonary bacterial infection originating from neutropenia. This condition has been revealed in other studies [43–45].

Another adverse effect is severe neck pain, which has also been reported in previous studies [15]. Zhou et al. observed that patients with new TAK onset or deteriorated neck pain had higher rates of constitutional symptoms and required higher GC dosage [15]. A significantly lower Hb concentration was also detected in such patients than in those without neck pain. Infections are more common in Chinese patients, but abnormal liver enzymes, new TAK onset or deteriorated neck pain appeared more frequently in other populations. No death was observed in our study.

This study has several limitations. First, the patients were only recruited from a single center. Hence, advanced studies are needed to verify the benefits of TCZ in contrast to traditional DMARDs.

## Conclusion

TCZ demonstrated obvious efficacy and safety in naïve-treated patients and patients with refractory TAK. TCZ could also induce a long period of remission. However, large-scale open-label and randomized trials are required to assess its long-term efficacy and safety and figure out an optimal scheduling.
